# In Vivo Activity of the Benzothiazinones PBTZ169 and BTZ043 against *Nocardia brasiliensis*

**DOI:** 10.1371/journal.pntd.0004022

**Published:** 2015-10-16

**Authors:** Norma Alejandra González-Martínez, Hector Gerardo Lozano-Garza, Jorge Castro-Garza, Alexandra De Osio-Cortez, Javier Vargas-Villarreal, Norma Cavazos-Rocha, Jorge Ocampo-Candiani, Vadim Makarov, Stewart T. Cole, Lucio Vera-Cabrera

**Affiliations:** 1 Laboratorio Interdisciplinario de Investigación Dermatológica, Servicio de Dermatología, Hospital Universitario “Dr. José Eleuterio González”, Monterrey, Nuevo León, México; 2 Centro de Investigación Biomédica del Noreste, IMSS, Monterrey, Nuevo León, México; 3 Departamento de Química Analítica de la Facultad de Medicina de la UANL, Monterrey, Nuevo León, México; 4 Bakh Institute of Biochemistry, Russian Academy of Science, Moscow, Russia; 5 Global Health Institute, École Polytechnique Fédérale de Lausanne, Lausanne, Switzerland; University of Tennessee, UNITED STATES

## Abstract

**Background:**

Mycetoma is a neglected, chronic, and deforming infectious disease caused by fungi and actinomycetes. In Mexico, *N*. *brasiliensis* is the predominant etiologic agent. Therapeutic alternatives are necessary because the current drug regimens have several disadvantages. Benzothiazinones (BTZ) are a new class of candidate drugs that inhibit decaprenyl-phosphoribose-epimerase (DprE1), an essential enzyme involved in the cell wall biosynthesis of Corynebacterineae.

**Methodology/Principal findings:**

In this study, the *in vitro* activity of the next generation BTZ, PBTZ169, was tested against thirty *Nocardia brasiliensis* isolates. The MIC_50_ and MIC_90_ values for PBTZ169 were 0.0075 and 0.03 μg/mL, respectively. Because *Nocardia* is a potential intracellular bacterium, a THP-1 macrophage monolayer was infected with *N*. *brasiliensis* HUJEG-1 and then treated with PBTZ169, resulting in a decrease in the number of colony-forming units (CFUs) at a concentration of 0.25X the *in vitro* value. The *in vivo* activity was evaluated after infecting female BALB/c mice in the right hind food-pad. After 6 weeks, treatment was initiated with PBTZ169 and its activity was compared with the first generation compound, BTZ043. Both BTZ compounds were administered at 100 mg/kg twice daily by gavage, and sulfamethoxazole/trimethoprim (SXT), at 100 mg/kg sulfamethoxazole, was used as a positive control. After 22 weeks of therapy, only PBTZ169 and SXT displayed statistically significant activity.

**Conclusion:**

These results indicate that DprE1 inhibitors may be useful for treating infections of *Nocardia* and may therefore be active against other actinomycetoma agents. We must test combinations of these compounds with other antimicrobial agents, such as linezolid, tedizolid or SXT, that have good to excellent in vivo activity, as well as new DprE1 inhibitors that can achieve higher plasma levels.

## Author Summary

Mycetoma is a neglected tropical disease caused by many etiological agents, including actinobacteria and true fungi. In Mexico, *Nocardia brasiliensis* and *Actinomadura madurae* account for more than 90% of the total cases. This subcutaneous infectious disease can affect skin and subcutaneous tissue; actinomycetomas are particularly osteolytic. The presence of abundant scar tissue, pus, and the intracellular growth of *Nocardia* make treatment very difficult. Current *N*. *brasiliensis* actinomycetoma therapy includes the use of trimethoprim-sulamethozaxole, diamino-diphenyl-sulphone (DDS), amikacin, and amoxicillin-clavulanate. *N*. *brasiliensis* is resistant to many other antimicrobials due in part to its richness in copies of genes related to pharmacoresistance, for instance *rpoB*, gyrase, beta-lactams, P450 cytochromes, etc. DprE1 inhibitors are new types of compounds that target a completely different gene, *dprE1*, encoding the decaprenylphosphoryl-d-ribose oxidase. Assays evaluating these experimental or other new drugs are necessary to develop a better therapeutic scheme for actinomycetoma, with more potent, less toxic antimicrobials.

## Introduction

*Nocardia brasiliensis* mycetoma is a subcutaneous infection characterized by tumefaction and the production of abscesses and fistulae. There are also microcolonies of the etiologic agent in pus. Mycetoma is also produced by fungi and a wide variety of actinomycetes, including *Nocardia*, *Actinomadura* and *Streptomyces* [1]. Because these etiologic agents originate from the soil, the dominant species in specific areas depend on the geographic location. In México, the majority of cases are caused by actinomycetales. Inflammation and scar tissue make it difficult for antimicrobials to penetrate and kill the bacteria. Several antimicrobials, including sulfonamides, aminoglycosides, and beta-lactams, have been used for the therapy of actinomycetoma [2]. However, in some cases, cure is not achieved, and because the disease is stigmatizing and disabling, it is important to evaluate new antimicrobials for use as therapeutic alternatives.

Benzothiazinones (1,3-benzothiazin-4-ones, BTZs) are a novel class of anti-mycobacterial agents that block the synthesis of decaprenyl-phospho-arabinose, the precursor of cell-wall arabinans [3]. Benzothiazinones have shown excellent activity against several Corynebacterineae genera, including *Corynebacterium*, *Mycobacterium*, *Rhodococcus* and *Nocardia*. BTZs are particularly active against *Mycobacterium tuberculosis*, displaying nanomolar minimal inhibitory concentrations (MIC), and are therefore more active than the existing tuberculosis drugs, including rifampin and isoniazid. The biochemical target of BTZs, the essential enzyme decaprenyl phosphoribose-2´-epimerase (DprE1), is commonly distributed among actinobacteria [4].

A new enhanced series of benzothiazinones, the PBTZs, have been produced by introducing a piperazine group into the scaffold [5]. Similar to BTZ043, the preclinical candidate PBTZ169 binds covalently to DprE1. The crystal structure of the *M*. *tuberculosis* DprE1-PBTZ169 complex revealed the formation of a semimercaptal adduct with Cys387 in the active site that may explain the irreversible inactivation of the enzyme [5].

Because of the close phylogenetic relationship among Corynebacterineae, it is possible that these anti-tubercular agents are also active against *Nocardiae*. In a previous study, we reported the *in vitro* activity of the early lead compound BTZ043 against *N*. *brasiliensis* [6]. Here, we analyze the susceptibility of 30 *N*. *brasiliensis* isolates from human mycetoma against the clinical candidate PBTZ169 using a microbroth dilution assay. We also tested the *ex vivo* activity of PBTZ169 in macrophage monolayers infected with *N*. *brasiliensis* and its *in vivo* activity in a BALB/c murine model of mycetoma.

## Methods

### Bacterial strains and culture conditions

In this study, we tested 30 isolates from the collection of the Laboratorio Interdisciplinario de Investigación Dermatológica (LIID) of the Servicio de Dermatología, Hospital Universitario, UANL, including *N*. *brasiliensis* HUJEG-1 (ATCC700358) previously used in other *in vitro* and *in vivo* assays [7, 8]. These isolates are from human mycetoma lesions and were identified by conventional biochemical methods as well as by sequence analysis of a portion of the 16S rRNA gene using the NOC-3 and NOC-4 primers [9].

### Drugs and chemicals

The BTZ043 and PBTZ169 were provided by two authors of the current study, VM and STC. Trimethoprim-sulfamethoxazole (suspension), at a concentration of 40 mg/200 mg, was obtained from Roche, New Jersey. D-7218 (tedizolid) was kindly donated by the Research Laboratory of the Dong-A Pharmaceutical Company (Yongin, South Korea). For animal studies, the SXT suspension was diluted in distilled water. BTZ043 and PBTZ169 were suspended in 0.25% hydroxy-propylmethyl-cellulose.

### MIC determination for BTZ169

We used the broth microdilution method based on the CLSI M24-A document that we previously described [10, 11]. As external controls, we used *Escherichia coli* ATCC 25922 and *Staphylococcus aureus* ATCC 29213. Because of the high susceptibility of *Nocardiae*, the concentrations ranged from 0.125 μg/mL to 0.0002 μg/mL.

### Preparation of a unicellular *Nocardia* suspension

Because *N*. *brasiliensis* grows as filaments, a unicellular suspension was prepared as published previously [12]. *N*. *brasiliensis* HUJEG-1 was cultured on Sabouraud agar (Difco Laboratories, Detroit, MI, USA) for 1 week and then sub-cultured in brain heart infusion (BHI; Difco, Labaratories) at 37°C in a shaker (New Brunswick Scientific C24, Edison, NJ, USA) at 110 rpm for 72 hrs. The bacterial mass was then separated by centrifugation (Eppendorf 5810R Hamburg Germany) and washed four times with saline. After grinding in an Evelham-Potter device (Fisher Scientific, Pittsburg, PA, USA), the suspension was centrifuged twice at 100 ×*g*; the supernatant was the unicellular suspension. The bacterial concentration was determined by plating on BHI agar with 5% sheep blood, and the suspension was stored in 20% glycerol at -70°C until use.

### THP-1 macrophage assays

The human monocyte cell line THP-1 (ATCC TIB-202)(American Type Culture Collection, Manassas, VA) was maintained in RPMI 1640 medium (Gibco-BRL, Gran Island, NY, USA) supplemented with 10% fetal calf serum (FCS; Gibco-BRL) and 1 mM sodium pyruvate (Sigma, St. Louis, MO, USA). To transform the cells into macrophages, the cells were sub-cultured four times without sodium pyruvate. The cell density was then determined in a hemocytometer, and the cell suspension was diluted as required in complete RPMI 1640 supplemented with 10% FCS and 6.25 ng/mL phorbol-12-myristate 13-acetate (Calbiochem Biosciences, Darmstadt, Germany) to obtain a density of 4 x 10^5^ cells/mL. A 1 mL aliquot of the cell suspension was seeded into each well of a 24-well microplate (Costar Corning, Daly City, USA), and the cell cultures were washed twice with RPMI 1640 every 48 h for no longer than 4 days.

### Determination of the intracellular activity of BTZ

The technique used has been published previously [12]. Briefly, a 3:1 multiplicity of infection (MOI) was used to determine the effect of antimicrobials on *Nocardia* intracellular growth. Two hours after infecting the monolayer, the medium was discarded and the monolayer was washed twice with warm PBS, pH 7.4. PBTZ was added at 0.25X, 1X, 4X and 16X the MIC in RPMI 1640 with 10% FCS and incubated for 6 h at 37°C in 5% CO_2_. We cannot use rifampin as an intracellular active control because *N*. *brasiliensis* is a naturally resistant bacteria. Instead, we used DA-7218, an oxazolidinone drug that previously demonstrated good intracellular and in vivo activity against *N*. *brasiliensis* [13]. The culture medium was discarded, and 1 mL of cold distilled water was added and incubated for 15 min. To release the intracellular bacteria, the monolayer was disrupted by pipetting up and down several times, and the suspension was collected in 1.5 mL Eppendorf tubes. *Nocardia* growth was quantified on BHI agar.

### Plasma quantitation of BTZs

To quantitate the plasma levels in mice, we administered the compounds to 8–12-week-old female BALB/c mice by gavage using BTZ043, PBTZ169 or SXT, all at 100 mg/kg. Blood samples from the periorbitary plexus were collected at 0, 20, 40, 60, 120, 240, 360, 480, and 600 min. The concentrations of BTZ 043, BTZ 169 and SXT were analyzed using a high-pressure liquid chromatography method developed in our laboratory.

To 50 μL of thawed plasma, 150 μL acetonitrile was added to precipitate the proteins. The mixture was vortexed for 1 minute and centrifuged for 5 min at 5304 × *g*, and the supernatant was filtered through 0.45 μm nylon filters (Waters, Milford, Mass.). Filtrates were collected into 150 μL inserts and analyzed by HPLC. Chromatographic separation was achieved using a HP1100 liquid chromatograph with a UV detector. A Synergi 4μPolar-RP 80A column 150 mm × 2 mm I.D., with a 4-μm particle size (Phenomenex, Torrance, CA) was used. Samples were eluted with a mobile phase consisting of 0.1% formic acid in water (solvent A) and 0.1% formic acid in acetonitrile (solvent B) in a 60:40 proportion. The flow rate of the mobile phase was 0.2 mL/min. The injection volume was 5 μL. Detection and quantification of benzothiazinones was performed by ultraviolet at a wavelength of 240 nm. The total run time was 10 min.

### Efficacy in mice

Eight- to twelve-week-old female BALB/c mice (Harlan Mexico S.A. de C.V., Mexico City) were infected with *N*. *brasiliensis* HUJEG-1. Experimental mycetoma was produced by injecting 20 mg (wet weight) of a *N*. *brasiliensis* suspension into the left hind footpad, as previously described [14]. Four weeks later, therapy was initiated. Groups of 15 animals were tested. One group of animals received saline solution by gavage as a negative control. The remainder were treated with PBTZ169, BTZ043, or SXT at 100 mg/kg administered twice daily by gavage for 10 weeks. The latter was used a positive control of experimental therapy. After 2 weeks of rest, the compounds were administered for a final period of 6 weeks. The effect of the drugs on the development of mycetomatous lesions was assessed by a blind reader using a previously published scale [14]. Potential differences among the groups against a control inoculated with saline solution were established using a variance test analysis.

### Ethics statement

The study was approved by the Comité Local de Investigación en Salud 1906, Centro de Investigación Biomédica del Noreste, IMSS, and the Comite de Investigacion, Facultad de Medicina, U.A.N.L. code DE11-002. The animal handling was performed according to the NORMA Oficial Mexicana NOM-062-ZOO-1999, (Especificaciones técnicas para la producción, cuidado y uso de los animales de laboratorio; Technical specifications for the production, care and handling of laboratory animals).

### Statistical procedures

We performed an ANOVA (analysis of variance) for the intracellular killing assay with THP-1 macrophages. For the animal assays, a test of variance was used. Likewise multiple comparisons tests were also performed using the statistical LSD (least significant difference). Because we lost some animals, the variance test was adjusted.

### *In silico* analysis

To verify the presence of orthologs of DprE1 in *Nocardia* species, we utilized the internet BLAST program to scan microbial genomes (http://blast.ncbi.nlm.nih.gov/Blast.cgi?PAGE_TYPE=BlastSearch&BLAST_SPEC=MicrobialGenomes), selecting *Nocardia* as organism database and *Mycobacterium tuberculosis* H37Rv DprE1 sequence (locus Rv3790) as the query. Because other complete genomes have been reported for actinomycetoma etiologic agents, we also used the MTB protein sequence to analyze the data from *Streptomyces somaliensis* (NCBI reference sequence WP_010471675.1) and *Actinomadura madurae* LIDD AJ290 (NCBI reference sequence WP_021594179.1).

## Results

### MIC values

The MIC values ranged from 0.03 μg/mL to 0.0037 μg/mL PBTZ169. The MIC_50_ and MIC_90_ values were 0.0075 and 0.030 μg/mL, respectively. The MIC for PBTZ169 for *N*. *brasiliensis* HUJEG-1 was 0.0037 μg/mL. The MICs of SXT, DA-7218, and BTZ043 for this strain were previously published [6, 13] and are 9.5/0.5, 8, and 0.125 μg/mL, respectively.

### Intracellular activity of BTZs

**Fig 1:**
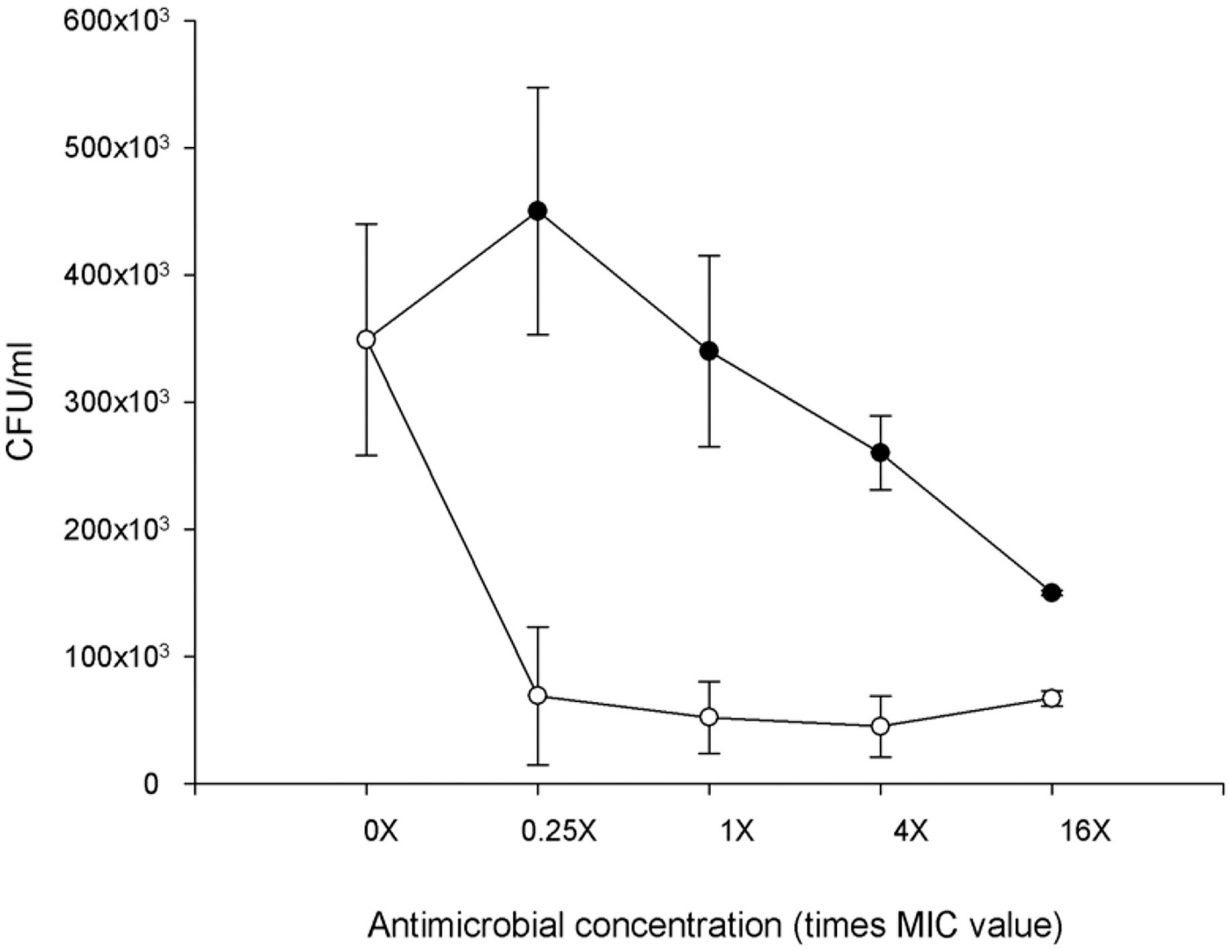
Intracellular activity of PBTZ169 against *Nocardia brasiliensis* HUJEG-1 after 8 h of replication inside THP-1 macrophages. The measurements were performed in triplicate. Each point represents the mean of the assays and error bars represent the standard deviations. There were significant differences at all concentrations for PBTZ169 (open diamonds) (*P*  =  0.021) (MIC = 0.0037 μg/mL). As a control, we used tedizolid (closed circles) (MIC = 8 μg/mL).

As shown in Fig 1, DA-7218 shows dose-dependent activity with statistical significance for the 4X and 16X concentrations. By contrast, PBTZ169 presents significant killing activity, even at 0.25X MIC. Statistically significant differences were observed for all PBTZ169 concentrations (*P* = 0.021) compared to both the negative control and DA-7218 (*P* = 0.0311).

### Plasma levels of drugs

**Fig 2:**
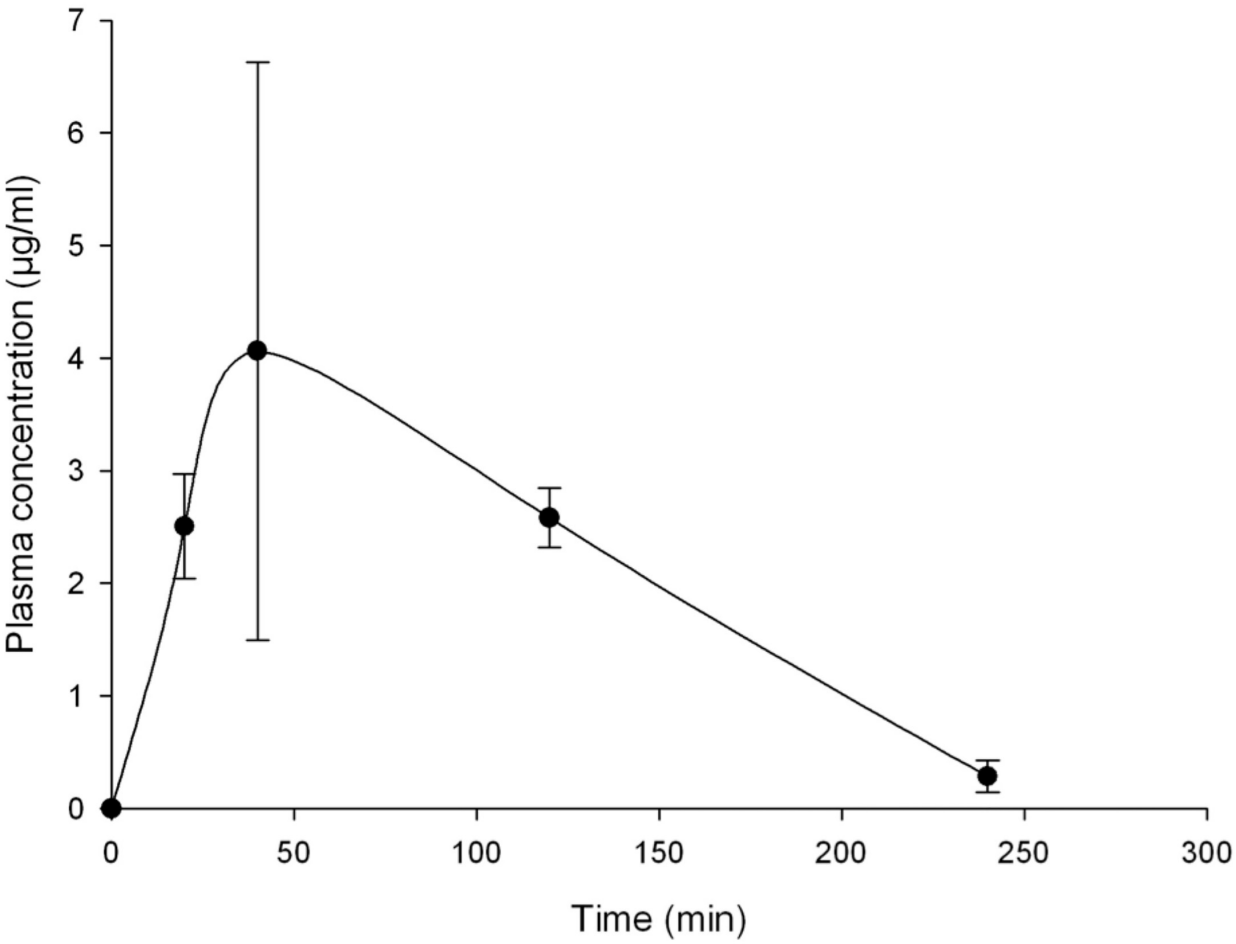
Plasma levels of BTZ043 observed in BALB/c mice. Animals were given SXT at 100 mg/kg by gavage. Each point represent the mean of three mice with bars representing the standard deviation obtained.

**Fig 3:**
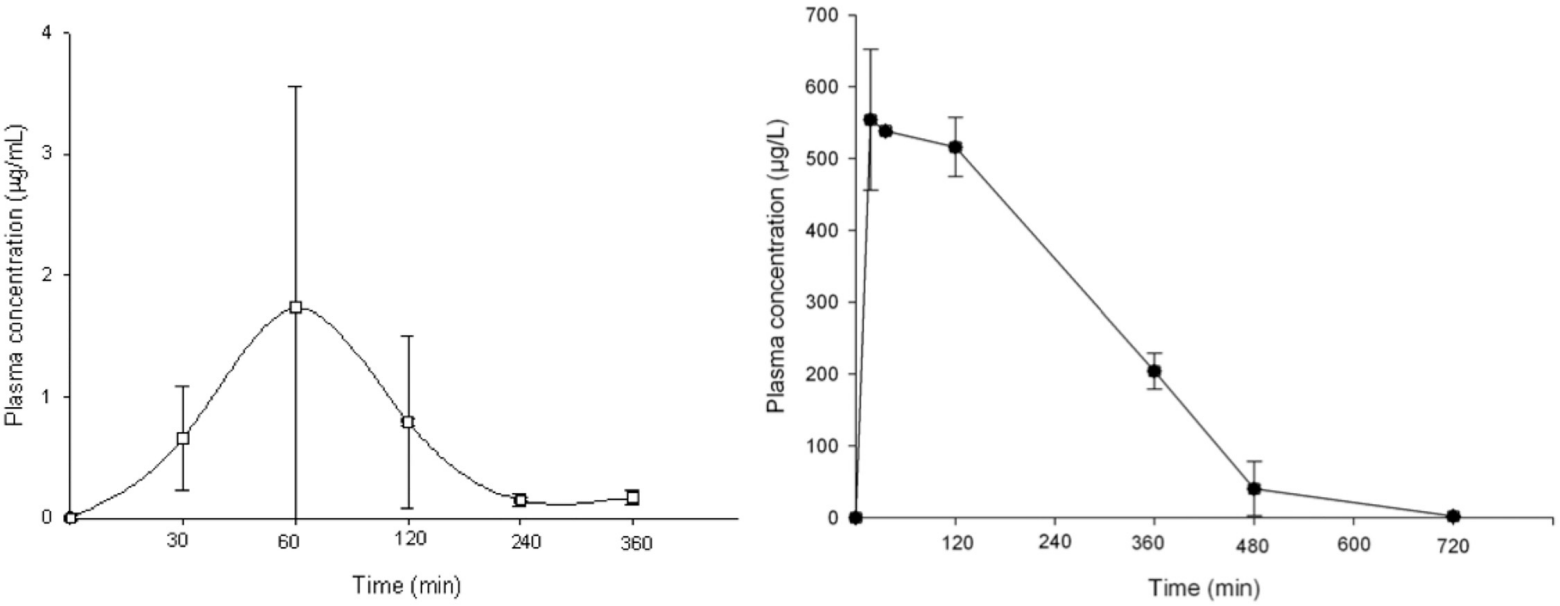
Plasma levels observed in BALB/c mice after applying, by gavage, PBTZ169 (left) and trimethoprim-sulfomethoxazole (right), both at 100 mg/kg. Three mice were bled and sacrificed at each point. Bars represent the standard deviation.

BTZ043 plasma levels in mouse were previously published [3]. At a dose of 100 mg/kg, it reaches a concentration of 4.06 μg/mL (Tmax of 40 min); which is quite similar to the levels in plasma observed in our case (Fig 2). In the case of PBTZ169, it presented a *Cmax* of 1.74 micrograms/mL, with a *Tmax* of 40 min (Fig 3). In Fig 3, we also present the plasma concentrations of SXT at 100 mg/kg, presenting a maximum concentration of 553.88 μg /mL 40 min after drug administration. The t ½ was 1.66 h, and the AUC was 1507.69 mg/L*h.

### Effect of BTZs in the mycetoma animal model

**Fig 4:**
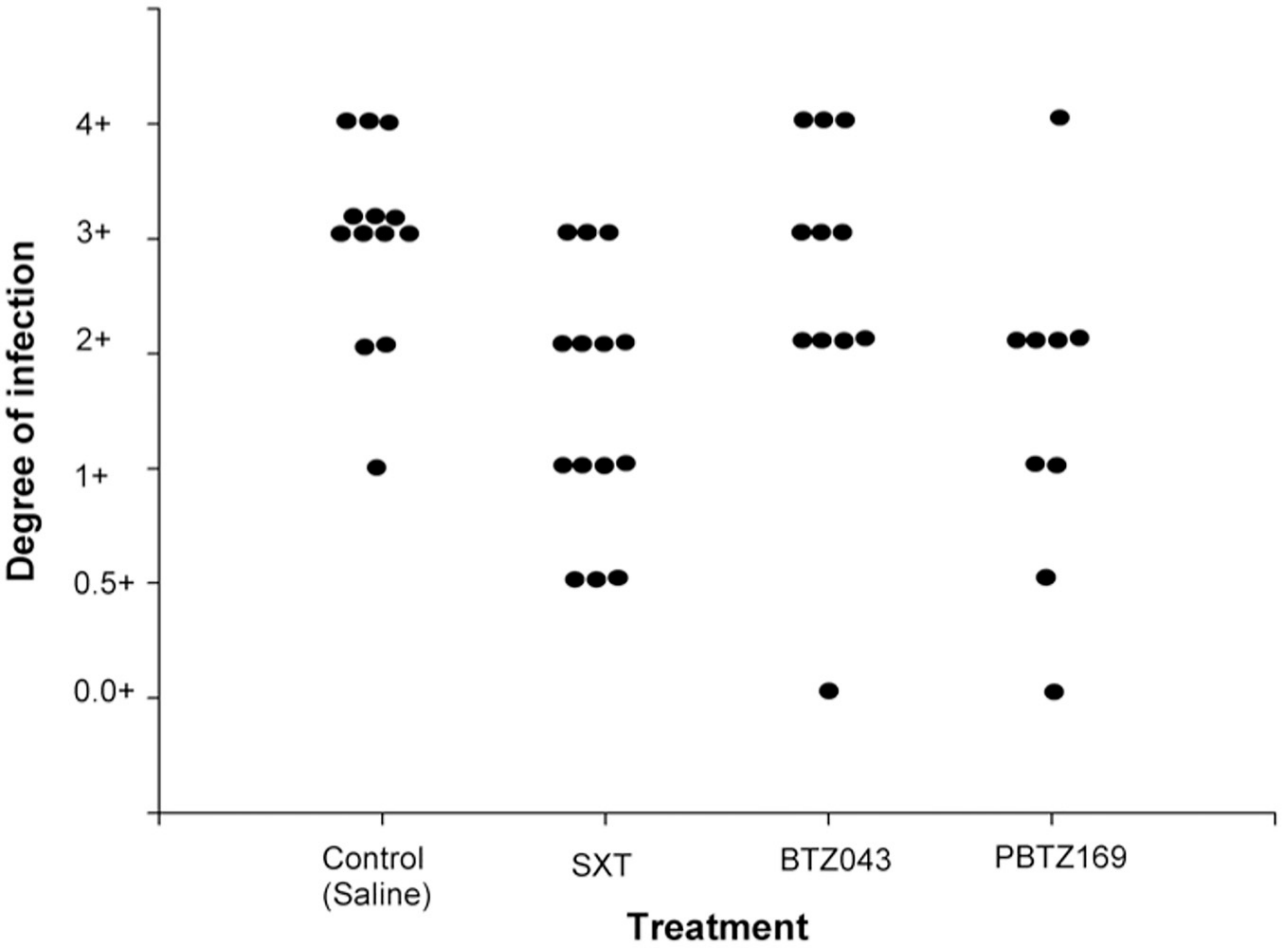
Effect of PBTZ169, BTZ043, and SXT on the development of mycetoma lesions in BALB/c mice infected with *N*. *brasiliensis* HUJEG-1. The Y axis is an arbitrary scale we developed to measure the degree of infection (14) and goes from the total absence of lesions (0.0 +) to the presence of abundant inflammation, abscesses and fistulae. Each dot represents the reading of one animal; all the groups were compared statistically against the saline control group by using the Variance test,. According to this analysis, significant differences were found for treatment with PBTZ169 (*P* = 0.017) but not BTZ043 (*P* = 0.667). The positive control group treated with SXT yielded a statistically significant value (*P* = 0.007).

When PBTZ169 and BTZ043 were administered at 100 mg/kg twice daily by gavage (Fig 4), only the former showed a statistically significant effect compared to the saline control (*P* = 0.017). No significant difference was detected in the BTZ043-treated group (***P*** = 0.667). The mouse group treated with SXT showed a statistically significant difference compared to the control group (*P* = 0.007).

### *In silico* analysis

**Fig 5:**
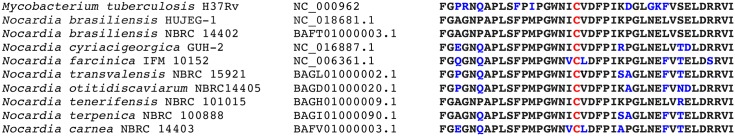
Alignment of *M*. *tuberculosis* DprE1 ortholog proteins in *Nocardia spp*. We observe that all species have orthologs with the susceptible genotype, a cysteine at position 368. This analysis included common *Nocardia* pathogens, such as *N*. *brasiliensis*, *N*. *cyryiacigeorgica*, *N*. *farcinica*, *N*. *transvalensis*, and *N*. *otitidiscaviarum*. We also included rarely pathogenic species, such as *N*. *tenerifensis*, *N*. *terpenica*, and *N*. *carnea*.

**Fig 6:**
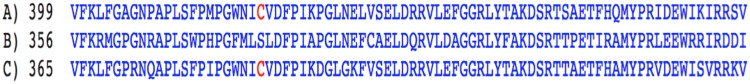
Protein sequence alignment of *M*. *tuberculosis* H37Rv DprE1 (A) and two orthologs in the genome of *N*. *brasiliensis* HUJEG-1. In red, we show Cys387. B: *N*. *brasilie*nsis YP_006807368 has 97% query cover and 62% identity to MTB DprE1. C: *N*. *brasiliensis* YP_006805098, presents a 99% query cover and 74% identity to MTB DprE1.

The susceptibility of *M*. *tuberculosis* DprE1 depends on the covalent binding of BTZ drugs to Cys387, and drug resistance results from the replacement of Cys387 by serine or alanine [5]. Our comparison of the sequence of DprE1 from *M*. *tuberculosis* with the genomes of *Nocardia* revealed that the majority of *Nocardia* spp. associated with human disease possesses *dprE1* orthologs with a cysteine at this position (Fig 5). Comparison of the *M*. *tuberculosis* DprE1 protein sequence with *N*. *brasiliensis* HUJEG-1 revealed the presence of two proteins, YP_006805098, with 99% query cover and 74% identity, and YP_006807368, with 97% query cover and 62% identity. However, the latter protein possesses a serine instead of a cysteine at position 368 (Fig 6). In *N*. *cyriacigeorgica* GUH-2 and N. *farcinica* IFM 10152, we found only one protein similar to MTB DprE1.

Actinomycetoma etiologic agents include other actinomycetales of the genera *Actinomadura*, (*A*. *pelletieri* and *A*. *madurae*), and *Streptomyces* (*S*. *somaliensis*). Because their genome sequences are available, except for *A*. *pelletieri*, we also searched for orthologous genes, and found only proteins with low identity, less than 30% of both the query cover and identity.

## Discussion

Benzothiazinones are highly potent drug candidates for the treatment of tuberculosis and other actinobacterial infections. Because of the nanomolar activity of benzothiazinones, we expected excellent *in vivo* activity. At 100 mg/kg twice daily, we observed a therapeutic effect, but only with PBTZ169. In *M*. *tuberculosis*, a microorganism with a thicker and more hydrophobic cell-wall than *N*. *brasiliensis*, BTZ043 at 50 mg/kg once daily resulted in a significant decrease in the lung and spleen bacterial burden [5]. PBTZ169 is a more effective drug, and in the mouse model of infection, it significantly decreases the amount bacilli at 25 mg/kg once daily compared with BTZ043 [3]. For *N*. *brasiliensis*, we used higher concentrations for experimental mycetoma (100 mg/kg, twice daily) to obtain a significant result. These results may be explained by the pharmacokinetic properties because the plasma levels reached by these drugs were relatively low, with a *Cmax* of 4 μg/mL and a *Tmax* of 20 min. However, previous studies showed that the PBTZ169 concentrations remain above the MIC for *M*. *tuberculosis* for nearly 24 hours following administration of a single dose [5], and this is also likely the case for *N*. *brasiliensis*.

The lower than expected activity of BTZ043 and PBTZ169 may also be because of the genetic nature of *N*. *brasiliensis*, a soil organism with a large chromosome of approximately 10 MB [15] that possesses a large amount of metabolic genes, including a second *dprE1* gene. Although BTZs and PBTZs are “suicide” substrates that react with the active site cysteine of DprE1, the second DprE1 enzyme in *N*. *brasiliensis* has a resistant genotype, with a Ser instead of a Cys in the position corresponding to 387. For rifampin and most quinolones, many *Nocardia spp*. are susceptible to these drugs, with the exception of *N*. *brasiliensis*, which is naturally resistant due to the presence of second *rpoB* and *gyrB* genes [16].

For *N*. *cyriacigeorgica* and *N*. *farcinica*, other important nocardial pathogens, only one gene was identified, which will likely make the use of these compounds in nocardiosis successful. Other actinomycetales that produce mycetoma, such as *Actinomadura* and *Streptomyces*, do not possess DprE1 orthologs, and may be resistant to this type of compounds, although the vitro susceptibility of these microorganisms must be tested.

Although PBTZ169 alone is highly active *in vivo* in both the acute and chronic models of murine tuberculosis, its activity is even better in combination with other drugs, particularly with bedaquiline and pyrazinamide, with better results than the standard of care treatment, including rifampin, isoniazid and pyrazinamide [5]. Multidrug therapy is essential for long-term, chronic infections, such as leprosy, tuberculosis and mycetoma, primarily to avoid the development of resistant strains. Future studies, both *in vitro* and *in vivo*, must be performed to determine the concentration for combinations of PBTZ169, possibly with SXT, linezolid or DA-7218 (tedizolid). This may result in new therapeutic schemes for the treatment of mycetoma caused by *N*. *brasiliensis*.
